# MALDI-TOF mass spectrometry identification of mosquitoes collected in Vietnam

**DOI:** 10.1186/s13071-022-05149-2

**Published:** 2022-01-28

**Authors:** Ly Na Huynh, Adama Zan Diarra, Hong Sang Nguyen, Long Bien Tran, Van Nguyen Do, Tran Duc Anh Ly, Van Hoang Ho, Xuan Quang Nguyen, Philippe Parola

**Affiliations:** 1 Aix Marseille Univ, IRD, AP-HM, SSA, VITROME, Marseille, France; 2 IHU-Méditerranée Infection, Marseille, France; 3 Institute of Malariology, Parasitology and Entomology Quy Nhon (IMPE-QN), Quy Nhon, Vietnam

**Keywords:** MALDI-TOF MS, Molecular identification, Mosquitoes, Vietnam

## Abstract

**Background:**

Matrix-assisted laser desorption/ionization time-of-flight mass spectrometry (MALDI-TOF MS) is a tool that has revolutionised clinical microbiology and has recently been described as an innovative and effective approach to arthropod identification.

**Methods:**

In this study, mosquitoes were captured in Vietnam using four different methods (human landing catch, CDC light traps, BG-Sentinel traps, animal-baited net traps). A total of 4215 mosquitoes were captured and morphologically identified as belonging to three genera: *Aedes*, *Anopheles* and *Culex*. We randomly selected 1253 mosquitoes, including 662 specimens of 14 *Anopheles* species, 200 specimens of two *Aedes* species and 391 morphologically unidentified *Culex* specimens, for molecular and MALDI-TOF MS analysis. The DNA from 98 mosquitoes (69* Anopheles* specimens, 23 *Culex* specimens and six *Aedes* sp. specimens) was subjected to molecular analysis, either to confirm our morphological identification or the MALDI-TOF MS results, as well as to identify the *Culex* species that were morphologically identified at the genus level and to resolve the discrepancies between the morphological identification and the MALDI-TOF MS identification.

**Results:**

High-quality MS spectra were obtained for 1058 of the 1253 specimens (84%), including 192/200 for *Aedes*, 589/662 for *Anopheles* and 277/391 for *Culex*. The blind test showed that 986/997 (99%) of the specimens were correctly identified by MALDI-TOF MS, with log score values ranging from 1.708 to 2.843. Eleven specimens of *Culex* could not be identified based on morphological features, MALDI-TOF MS or molecular analysis.

**Conclusions:**

This study enabled us to identify several species of mosquitoes from Vietnam using MALDI-TOF MS, and to enrich our database of MALDI-TOF MS reference spectra.

**Graphical Abstract:**

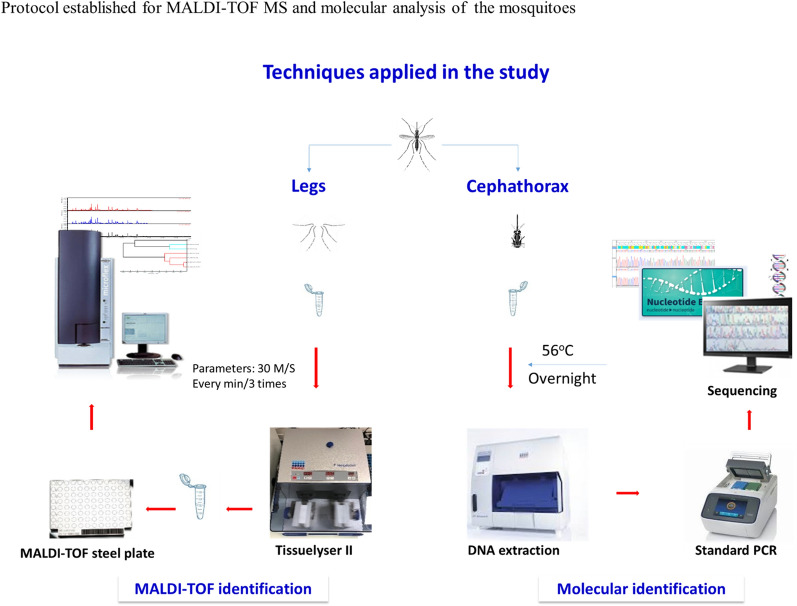

**Supplementary Information:**

The online version contains supplementary material available at 10.1186/s13071-022-05149-2.

## Background

Mosquitoes are one of the most important insect species in the world and approximately 3500 species are currently recognised [1]. They pose a major threat to human public health and economic development due to their ability to transmit and spread infectious pathogens, such as those causing malaria, dengue fever [dengue virus (DENV)], yellow fever [yellow fever virus (YFV)], Japanese encephalitis [Japanese encephalitis virus (JEV)], chikungunya [chikungunya virus (CHIKV)] and filariasis [2]. Malaria, caused by *Plasmodium* malarial parasites (i.e. *P. falciparum*, *P. vivax*, *P. malariae*, *P. ovale *and *P. knowlesi*), is transmitted to humans by female *Anopheles* spp. mosquitoes [3–5]. The WHO estimated that there were 229 million cases of malaria in 2019, resulting in 409,000 deaths worldwide [6]. Dengue infection, caused by any of the four DENV serotypes (DENV-1, -2, -3, and -4), is transmitted to humans via the bites of infected female *Aedes aegypti* or *Aedes albopictus* mosquitoes [7]. Dengue is epidemic in 128 countries, with four billion people living in areas at risk of dengue infection [8]. There are 100 million cases of dengue annually around the world, resulting in approximately 20,000 deaths [9].

In Vietnam, 255 mosquito species have been described, belonging to 42 subgenera and 21 genera [10]. The majority fall into three genera, namely *Aedes*, *Anopheles* and *Culex*, and include vectors of agents of infectious pathogens [11]. The number of species being discovered is steadily increasing, some of which are difficult to distinguish from others or are morphologically indistinguishable using traditional systematic methods [12]. Molecular biology techniques, such as enzyme electrophoresis and PCR analyses, have been used to discriminate between homogeneous species [13, 14]. However, these techniques are limited due to being time-consuming and expensive and by requiring amplified gene sequences, which are not available in the GenBank databases for certain species [15, 16].

Recent studies show that matrix-assisted laser desorption/ionization time-of-flight mass spectrometry (MALDI-TOF MS) may be a rapid and accurate tool for the identification of arthropods, their blood meal sources and potentially associated microorganisms [17–19]. Compared to sequencing, MALDI-TOF MS is an easy-to-use, time- and cost-saving technique in entomology, especially if a machine that is generally purchased for microbiology is available [20, 21]. It is a technique based on the identification of proteins contained in a sample of interest. The mass/charge ratio of each protein is measured as it crosses an electromagnetic field after propulsion of the protein molecules by an ultraviolet laser. The mass/charge of the proteins thus measured generates a specific protein mass spectrum of the sample known as the “protein signature”. This spectrum is then compared to a reference database containing the spectra of species which have been formally identified morphologically and molecularly [21]. In medical entomology, the choice of the body part of the arthropod and the method of preservation are parameters that can influence the quality of the spectrum [17]. However, the choice of the body part to be used for MALDI-TOF MS analysis depends on the type of arthropod, such as the legs for mosquitoes and ticks [20, 22, 23], the head for bedbugs [24] and the cephalothorax for lice [25]. This proteomic technique has overcome the limitations of traditional morphological and molecular identification methods because it does not require specific entomological knowledge and considerably reduces the time and cost involved [14, 21, 26].

The aim of the present study was to apply MALDI-TOF MS to identify some mosquito species collected in Vietnam in order to first create a reference database for rapid and accurate characterisation.

## Methods

### Mosquito collection and morphological identification

**Fig. 1:**
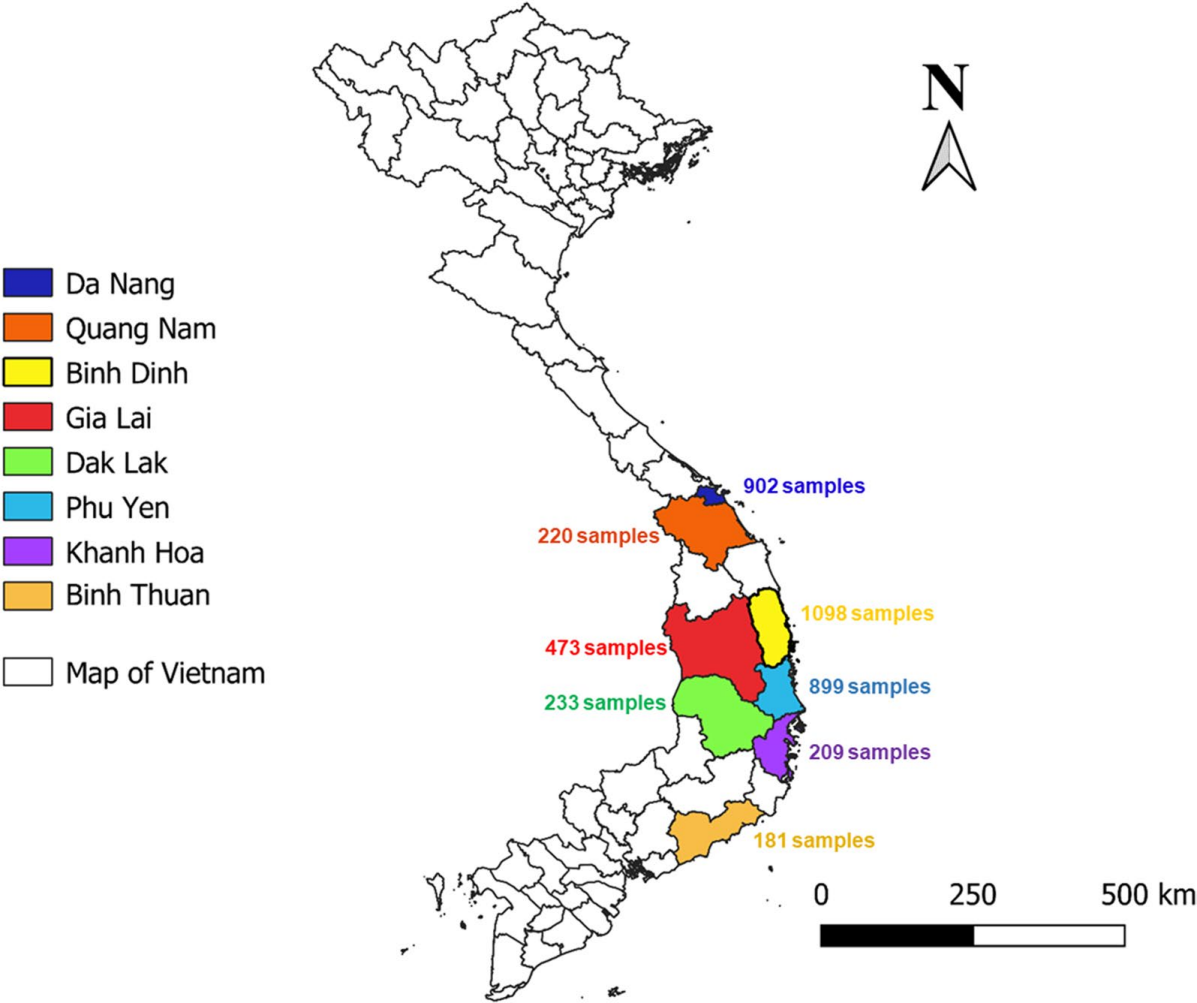
Location of study sites in the Central Highlands of Vietnam

All mosquitoes were collected by an entomological team from the Institute of Malariology, Parasitology and Entomology in Quy Nhon, Vietnam (IMPE-QN), at the beginning of the rainy season (May) and the beginning of the dry season (January) between 2018 and 2020 in the Central Highlands of Vietnam. The collection area included eight provinces: Hoa Vang (16°03′N; 108°01′E) District, Da Nang Province; Nam Giang (15°65′N; 107°50′E) District, Quang Nam Province; Van Canh (13°37′N; 108°59′E) District, Binh Dinh Province; Dong Xuan (13°22′N; 109°02′E) District, Phu Yen Province; Ea Kar (12°49′N; 108°27′E) District, Dak Lak Province; Krong Pa (13°15′N; 108°45′E) District, Gia Lai Province; Khanh Vinh (12°16′N; 108°53′E) District, Khanh Hoa Province; and Ham Thuan Nam (11°09′N; 108°03′E) District, Binh Thuan Province (Fig. 1).

Collections were performed on eight consecutive nights/days using four different methods: human landing catch (HLC), CDC light trap (CDC-LT), animal-baited net trap and Biogents Sentinel mosquito trap (BG-Sentinel trap; BioGents AG, Regensburg, Germany). Collections took place in the forests, homes and plot huts and around local people’s homes in order to capture mosquitoes representing a greater number of genera and species. HLCs were used for night/day collection both indoors and outdoors. The mosquito collectors were local adults who volunteered to capture mosquitoes using HLCs and subsequently trained as mosquito collectors. They were regularly observed for signs of malaria or dengue and were treated if infected. A group of four people worked from 6:00 pm until 12:00 pm sampling *Anopheles* spp. mosquitoes, and from 5:30–7:30 am to 5:30–7:30 pm sampling *Aedes* spp. mosquitoes. Four CDC-LTs and two BG-Sentinel traps were also used for indoor and outdoor overnight captures. They were positioned outdoors with a minimum between-trap distance of 100 m. Indoor traps were placed near bed nets around human sleeping areas. All traps were suspended in the areas targeted from 6:00 pm until 6:00 am. The animal-baited net traps were positioned from 6:00 pm until 6:00 am.

*Anopheles* and *Aedes* mosquitoes were identified morphologically at the species level and *Culex* mosquitoes were identified at the genus level using Vietnamese mosquito identification keys [27–30]. Only female mosquitoes were used in this study, and captured specimens were stored in silica gel (Carl Roth GmbH, Karlsruhe, Germany) for the most part, or frozen at − 20 °C, before being sent to the Institut Hospitalo-Universitaire (IHU) Méditerranée Infection in Marseille, France for analysis. Samples were stored and subsequently analysed over a period of between 1 and 2.5 years.

### Molecular identification of mosquitoes and phylogenetic analysis

Total DNA was extracted using a BioRobot EZ1 instrument (QIAGEN, Hilden, Germany) according to the manufacturer’s instructions. Cephalothoraxes from individual mosquitoes were placed in tubes containing 180 µl of G2 buffer and 20 µl of proteinase K and the samples incubated at 56 °C overnight. DNA extraction was performed from 200 µl of the incubation solution, eluted in 100 μl and stored at − 20 °C.

The DNA from 98 mosquitoes, including 43 mosquitoes the spectra of which were used to create our MALDI-TOF MS database and 55 mosquitoes selected for quality control of our MALDI-TOF MS identification, was subjected to molecular identification. The group of 55 mosquitoes selected for quality control of the MALDI-TOF MS identification method consisted of 34 mosquitoes whose identification by MALDI-TOF MS agreed with that by our morphological identification and 21 mosquitoes whose identification by MALDI-TOF MS showed discrepancies with that by our morphological identification. Standard PCR that specifically amplified a partial 720-bp cytochrome C oxidase I (*COI*) gene [forward primer *COI*_1 (LCO1490): 5′-GGT CAA ATC ATA AGA TAT TGG-3′; reverse primer (HC02198): 5′-TAA ACT TCA GGG TGA CCA AAA AAT CA-3′] was carried out and followed by sequencing [31]. We also sequenced the 720-bp *COI* fragment of 34 specimens of different species of mosquitoes to confirm our MALDI-TOF MS identification using the same primers. Samples for which we did not successfully sequence the *COI*_1 gene were then subjected to another standard PCR and sequencing using the gene known as *COI*_2 [forward primer (CI-J-1632): 5′-TGATCAAAAAATTTATAAT-3′; reverse primer (CI-N-2191): 5′-GGGGTAAAAAAAATATAAACTTC-3′] amplifying a 560-bp partial sequence [32]. We used the acetylcholinesterase 2 (*Ace2*) gene [direct primer (ACEpip): 5′-GGAAAACAAACGACGT ATGTACT-3′; reverse primer (B1246s): 5′-TGGAGCCTCCTCTTCACGGG-3′] amplifying a 610-bp fragment of *Culex pipiens* and a 274-bp fragment of *Culex quinquefasciatus* to distinguish the two species [33].

For specimens of *Anopheles maculatus* sensu lato (s.l.) that were not identified by MALDI-TOF MS, we performed standard PCR and sequencing targeting a partial 459-bp internal transcribed spacer 2 (*ITS2*) [forward primer (5.8F): 5′-TGTGAACTG CAGGACACATG-3′; reverse primer (28R): 5′-ATGCTTAAATTTAGGGGGTA-3′] [34].

Standard PCR and sequencing were performed as described in previous studies [23]. Negative controls (single PCR mix and sterile water) and positive controls (DNA extracted from identified *Anopheles gambiae* and *Ae. albopictus* from our laboratory colony) were included in each PCR run. The sequences obtained were assembled and analysed using ChromasPro software (version 1.77) (Technelysium Pty Ltd., Tewantin, Australia) and submitted for analysis to the NCBI BLAST website (http://blast.ncbi.nlm.nih.gov). The phylogenetic tree was built using Mega 7.0 software (https://www.megasoftware.net) [35]. Analyses were performed using the maximum likelihood method proposed by MEGA 7 and bootstrap analyses were performed using 500 replicates.

### Preparation of mosquitoes for MALDI-TOF MS analysis

Mosquitoes with at least three legs were selected for the MALDI-TOF MS analysis. Each mosquito was treated individually by grinding the legs in a 1.5-ml Eppendorf tube containing 15 μl of 70% (v/v) formic acid (Sigma-Aldrich, St. Louis, MO, USA) and 15 μl of 50% (v/v) acetonitrile (Fluka, Buchs, Switzerland), with 1.0-mm-diameter glass beads (Sigma France, Lyon, France) using a tissue lyser machine (Qiagen); the optimal parameters had been previously established [36]. The crushed legs were centrifuged, and a 1-μl aliquot of the supernatant of each sample was deposited in quadruplicate on a MALDI-TOF MS steel plate (Bruker Daltonics, Wissembourg, France) and covered after drying with a matrix solution composed of 1 μl of saturated alpha-cyano-4-hydroxycinnamic acid (Sigma France), 50% acetonitrile (v/v), 2.5% trifluoroacetic acid (v/v) (Sigma-Aldrich Co. Ltd., Gillingham Dorset, UK) and high-performance liquid chromatography-grade water [19, 37]. The target plate was then air-dried for a few minutes at room temperature before being introduced into the Microflex LT MALDI-TOF MS apparatus (Bruker Daltonics) for analysis. The quality of the matrix, the sample loading and the performance of the MALDI-TOF MS apparatus were controlled using the leg of an *Ae. albopictus* from our laboratory as a positive control.

### MALDI-TOF MS parameters

The spectral profiles obtained from the mosquito legs were visualised using a Microflex LT MALDI-TOF mass spectrometer with FlexControl software (version 3.3; Bruker Daltonics). The spectra were acquired in a positive linear mode at a laser frequency of 50 Hz. The accelerating voltage was 20 kV, and the extraction delay time was 200 ns. Each spectrum corresponded to the ions obtained from the 240 laser shots performed in six regions at the same location and acquired automatically using the AutoXecute Flex Control software (version 2.4; Bruker Daltonics). The MS profiles were visualised using the FlexAnalysis v3.4 software, MALDI Biotyper Compass Explorer v4.1.70 (Bruker Daltonics), and ClinProTools v3.0 software (Bruker Daltonics) was used for data processing.

### Spectral analysis and reference database creation

All spectra were visualised using the FlexAnalysis v.3.3 software to check their quality, reproducibility, and specificity. Spectra of poor quality, i.e. those which had low intensity (< 3000 AU), which were non-reproducible and which featured background noise, were excluded from the study. Finally, cluster analysis was performed to visualise the reproducibility and specificity of the spectra using the Main Spectrum Profile (MSP) dendrogram function of the MALDI Biotyper Compass Explorer v4.1.70 software. The dendrogram was performed using two to three spectra per species, excluding *Anopheles barbirostris* and *Culex pallidothorax*, for which there was only one spectrum for each species. The MS dendrogram is the result of comparing the MSPs produced by the MALDI Biotyper software and clustered according to the mass profile of the proteins (i.e. their mass signals and intensities), and this MS dendrogram illustrates how the samples are related to one another. The MS dendrograms helped us select representative reference spectra of the species [23]. Between one and four reference spectra per species were then added to our in-house arthropod database of MS reference spectra [20]. However, the reference spectra of *Anopheles kochi* and *Anopheles jeyporiensis* were not added to the database as the identification of *An. kochi* had not been molecularly confirmed and the spectrum of *An. jeyporiensis* was of poor quality.

### Blind test for mosquito identification

The MS spectra from *Ae. albopictus*, *Ae. aegypti* and *Culex* spp. were blind tested against our pre-existing database containing spectra from *Ae. albopictus*, *Ae. aegypti*, *Cx. quinquefasciatus* and *Culex sitiens* species, the reference spectra of which were already available in our MALDI-TOF MS database. For specimens of *Culex* spp. that were not identified by this first blind test and the *Anopheles* species, a blind test was performed with the remaining spectra after creating a database of reference spectra of the different species confirmed by molecular biology. A total of 43 specimens, including between one and four of each species, were used to create the MALDI-TOF MS spectral database of Vietnamese mosquitoes. The level of reliability of species’ identification was determined using log score values (LSVs) of between 0 and 3 using the MALDI-TOF Biotyper software, which corresponded to a degree of similarity between the queried spectra and the reference spectrum in the database. The LSV threshold score set for correct identification of mosquito species was 1.70 in this study as this is the optimal threshold value applied for arthropod identification using MALDI-TOF MS [38, 39].

## Results

### Mosquito collection and morphological identification

A total of 4215 individual mosquitoes were captured using different methods, including 1301 specimens (30.9%) by HLCs, 1335 (31.7%) by CDC-LTs, 891 (21.1%) by animal-baited net traps and 688 (16.3%) by BG-Sentinel traps. Mosquitoes collected by CDC-LTs, HLCs and animal-baited net traps were more abundant in quantity and number of species than those caught using the BG-Sentinel traps. The number of specimens collected per province was: Da Nang (*n* = 902), Quang Nam (*n* = 220), Binh Dinh (*n* = 1098), Gia Lai (*n* = 473), Dak Lak (*n* = 233), Phu Yen (*n* = 899), Khanh Hoa (*n* = 209) and Binh Thuan (*n* = 181) (Table 1; Fig. 1; Additional file 1: Table S1). Two *Aedes* species were identified morphologically: *Ae. aegypti* (*n* = 507; 12%) and *Ae. albopictus* (*n* = 640; 15.2%). Fourteen *Anopheles* species were identified based on morphological characteristics: *Anopheles aconitus* (*n* = 15; 0.4%), *Anopheles annularis* (*n* = 3; 0.1%), *Anopheles barbirostris* (*n* = 7; 0.2%), *Anopheles dirus* s.l. (*n* = 281; 6.7%), *An. kochi* (*n* = 8; 0.2%), *An. maculatus* s.l. (*n* = 1062; 25.2%), *Anopheles minimus* s.l. (*n* = 183; 4.2%), *An. jeyporiensis* (*n* = 1; 0.02%), *Anopheles peditaeniatus* (*n* = 81; 1.9%)*, Anopheles sinensis* (*n* = 11; 0.2%), *Anopheles splendidus* (*n* = 36; 0.8%)*, Anopheles jamesii* (*n* = 49; 1.2%), *Anopheles vagus* (*n* = 35; 0.8%) and *Anopheles varuna* (*n* = 9; 0.2%). The *Culex* mosquitoes, representing 1287 mosquitoes (30.7%), were not identified to the species level when specific morphological characters could not be identified with certitude due to being damaged during collection, transport or storage.

**Table 1:** The number and composition of the mosquitoes collected by different methods in Vietnam between May 2018 and January 2020

Morphological ID	Different collection methods									
	Human-landing catch		Animal-baited net trap		CDC-LT		BG-Sentinel trap		Total	
	No. collected	%	No. collected	%	No. collected	%	No. collected	%	Total number collected	Total %
*Aedes albopictus*	403	9.6	0	0	0	0	237	5.6	640	15.2
*Ae. aegypti*	261	6.2	0	0	0	0	246	5.8	507	12
*Anopheles aconitus*	3	0.1	5	0.1	7	0.2	0	0	15	0.4
*An. annularis*	0	0	2	0.1	1	0.02	0	0	3	0.1
*An.barbirostris*	0	0	3	0.1	4	0.1	0	0	7	0.2
*An. dirus*	187	4.4	23	0.6	71	1.7	0	0	281	6.7
*An. kochi*	0	0	3	0.1	5	0.1	0	0	8	0.2
*An. jamesii*	12	0.3	12	0.3	25	0.6	0	0	49	1.2
*An. jeyporiensis*	0	0	1	0.02	0	0	0	0	1	0.02
*An. maculatus*	323	7.7	289	6.9	450	10.7	0	0	1062	25.2
*An. minimus*	83	1.9	34	0.8	66	1.5	0	0	183	4.2
*An. peditaeniatus*	0	0	45	1.1	36	0.8	0	0	81	1.9
*An. sinensis*	0	0	6	0.1	5	0.1	0	0	11	0.2
*An. splendidus*	8	0.2	22	0.5	6	0.1	0	0	36	0.8
*An. vagus*	7	0.2	26	0.6	2	0.1	0	0	35	0.8
*An. varuna*	3	0.1	4	0.1	2	0.04	0	0	9	0.2
*Culex* spp.	11	0.3	416	10	655	15.5	205	4.9	1287	30.7
Total	4215									

### MALDI-TOF MS identification of the mosquitoes

**Fig. 2:**
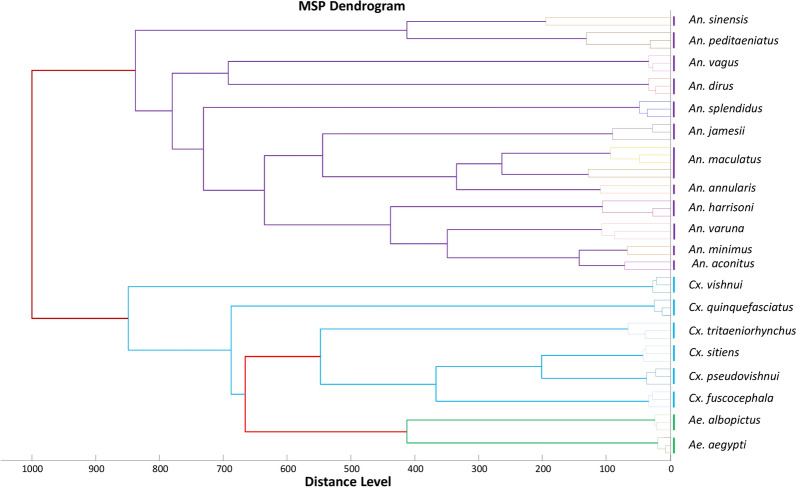
Dendrogram constructed from between two and three representative leg spectra of different mosquito species collected in Vietnam between May 2018 and January 2020. The dendrogram was created using Biotyper v3.0 software and the distance units correspond to the relative similarity of the MS spectra. The red lines show the cut-off level between mosquito genera. The purple lines show the cut-off level between the different *Anopheles* species. The blue and green lines show the cut-off between *Culex* and *Aedes* species, respectively

Of the 4215 mosquitoes collected, 1253 (30%) mosquitoes, including 662 specimens of *Anopheles* spp., 391 specimens of *Culex* spp. and 200 specimens of *Aedes* spp., were randomly selected for MALDI-TOF MS analysis (Table 2). Overall, good-quality MS spectra were obtained from the legs of 1058 of the 1253 (84%) mosquitoes subjected to MALDI-TOF MS analysis. The percentage of good-quality spectra was highest for *Aedes* spp., with 96% (192/200) good-quality spectra, followed by *Anopheles* spp. (88.97%; 589/662) and *Culex* spp. (70.84%; 277/391). Visually, the comparison of MS profiles of legs of different species using FlexControl analysis revealed intra-species reproducibility and inter-species specificity (Additional file 2: Fig. S1). The MS protein profiles of between two and three specimens of each mosquito species used to generate an MSP dendrogram showed a clustering of specimens of the same species on the same branch (Fig. 2). The average discriminant peak intensity of 19 mosquito species, excluding those with low numbers, is presented in Additional file 3: Table S2.

**Table 2:** Number of specimens of each morphologically identified mosquito species randomly selected for MALDI-TOF MS analysis, creation of the reference database and blind test results for MS identification and log-score values of each species

Morphological ID	No. tested/no. collected	No. of good spectra	No. added to database	MALDI-TOF MS ID	LSV range (minimum–maximum)
*Ae. albopictus*	100/640	94	0	*Ae. albopictus*^a^ (94)	1.892–2.371
*Ae. aegypti*	100/507	98	0	*Ae. aegypti*^a^ (98)	1.729–2.39
*An. aconitus*	10/15	7	2	*An. aconitus* (5)	1.732–2.786
*An. annularis*	2/3	2	1	*An. annularis* (1)	2.082
*An. barbirostris*	3/7	1	1	*An. barbirostris* (0)	Not applicable
*An.dirus*	79/281	79	3	*An. dirus* (75)	1.78–2.782
*An. kochi*	7/8	0	0	Not identified	Not applicable
*An. jamesii*	36/49	34	4	*An. jamessi* (29)	1.844–2.605
*An. jeyporiensis*	1/1	0	Not applicable	Not applicable	Not applicable
*An. maculatus*	329/1062	287	2	*An. maculatus* (276)	1.708–2.782
*An. minimus* s.l.	79/183	7	1	*An. minimus* (4)	2.174–2.815
		71	3	*An. harrisoni* (66)	1.836–2.821
*An. peditaeniatus*	46/81	43	4	*An. peditaeniatus* (37)	1.814–2.539
*An. sinensis*	10/11	8	1	*An. sinensis* (7)	1992–2.755
*An. splendidus*	36/36	28	2	*An. splendidus* (24)	1.851–2.449
*An. vagus*	16/35	14	3	*An. vagus* (11)	2.088–2.777
*An. varuna*	8/9	8	1	*An. varuna* (7)	2.065–2.449
*Culex* sp.	391/1287	277	0	*Cx. quinquefasciatus*^a^ (48)	1.725–2.401
*Culex* sp.			4	*Cx. tritaeniorhynchus* (58)	1.739–2.338
*Culex* sp.			4	*Cx. pseudovishnui* (46)	1.820 –2.012
*Culex* sp.			1	*Cx. pallidothorax* (0)	0
*Culex* sp.			2	*Cx. fuscocephala* (25)	1.756–2.843
*Culex* sp.			4	*Cx. vishnui* (44)	1.72–2
*Culex* sp.			0	*Cx. sitiens*^a^ (30)	1.737–2.144
*Culex* spp.			Not applicable	Not identified	Not applicable
Total	1253/4215	1058	43		

^a^Species was already available in our database

( ) the values in parentheses: the number of specimens of each species was identified using MALDI-TOF MS

**Fig. 3:**
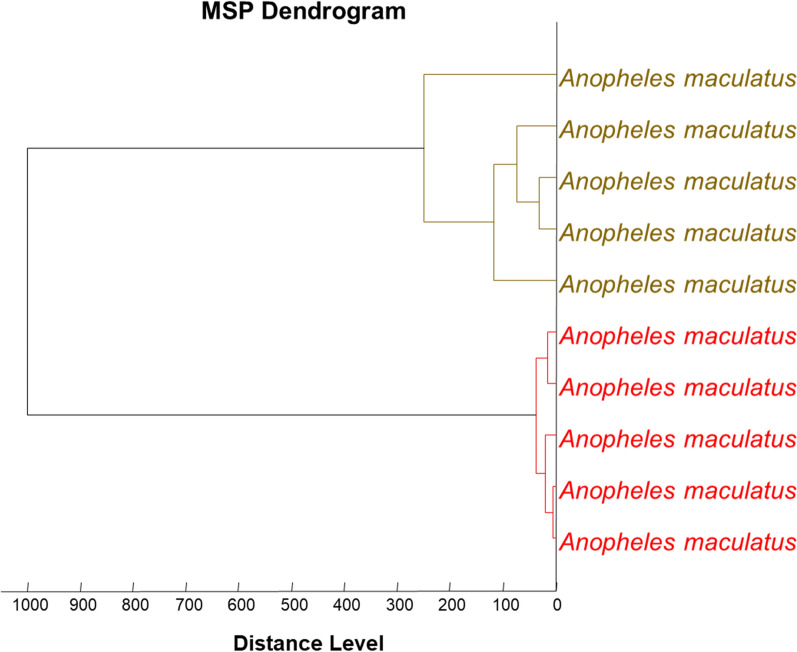
Dendrogram revealing that the spectra of mosquitoes identified as *Anopheles maculatus* senus lato formed two distinct branches

Querying 192 good-quality spectra of *Aedes* spp. against our in-house MALDI-TOF MS database showed that 94 spectra were *Ae. albopictus* and 98 were *Ae. aegypti*, with LSVs ranging from 1.729 to 2.39 (Table 2). For *Anopheles* spp., we added 28 reference spectra from between one and four spectra of each species, confirmed by molecular biology analysis, to our MALDI-TOF MS database, which did not contain spectra of the concerned species. The remaining 561 spectra were then blind tested against the updated database, revealing that 96.8% (543/561) had been correctly identified, i.e. presented a match between our morphological identification and MALDI-TOF MS results. The LSVs of these *Anopheles* spp. specimens ranged from 1.708 to 2.821 (Table 2). For the remaining 18 specimens (3.2%) there were discrepancies between our morphological identification and the MALDI-TOF MS identification. Molecular biology analysis confirmed the MALDI-TOF MS identification of all species, with the exception of specimens of *An. maculatus* s.l. The dendrogram visually produced using the spectra of mosquitoes identified as *An. maculatus* s.l. shows that they form two distinct branches (Fig. 3).

Querying the 277 legs of *Culex* spp. against our database, which contained the spectra of 17 *Culex* species, showed that 28.2% (78/277) were identified, including 48 *Cx. quinquefasciatus* and 30 *Cx. sitiens*, with LSVs ranging from 1.725 to 2.401, and from 1.737 to 2.144, respectively. From the remaining 199/277 legs of *Culex* spp. which were unidentified, we randomly selected 15 of the 199 *Culex* specimens to create our database with reference spectra of four *Culex tritaeniorhynchus*, four *Culex pseudovishnui*, four *Culex vishnui* s.l., two *Culex fuscocephala*, and one *Culex pallidothorax* identified by molecular biology. The second blind test of the remaining 184 spectra against the updated database showed that 92.4% (170/184) were identified, with LSVs ranging from 1.739 to 2.843. Of the 173 *Culex* mosquitoes which were identified, 58 specimens were *Cx. tritaeniorhynchus*, 46 were *Cx. pseudovishnui*, 44 were *Cx. vishnui* s.l. and 25 were *Cx. fuscocephala* (including 3 specimens which showed discrepancies between the morphological and the MALDI-TOF MS results, which were subsequently confirmed by molecular analysis). Finally, 11 specimens with good spectra were not identified by MALDI-TOF MS and could not be identified molecularly because we could not sequence them using the three types of genes (Additional file 4: Fig. S2).

### Molecular identification of the mosquitoes

Sequencing and BLAST of the *COI*_1 or *COI*_2 gene sequences from the *Anopheles* and *Aedes* species selected for the database revealed reliable and consistent species’ identification according to the morphological identification, with between 98.4 and 100% identity with GenBank sequences (Table 3). The *COI*_1 gene was used to identify species of 15 *Culex* spp. randomly selected to enter in our database. BLAST showed that four were between 99.3% and 100% identical to *Cx. tritaeniorhynchus* (MF179219; MF179221; MF179231; MF179232), four were between 97.9% and 98.2% identical to *Cx. pseudovishnui* (AB738092; LC054483), four were between 99.2% and 100% identical to *Cx. vishnui* (MF179240), two were 100% identical to *Cx. fuscocephala* (HQ398887) and one was 98% identical to *Cx. pallidothorax* (MF179281). Only one *Cx. quinquefasciatus* species could not be identified using *COI*_1 DNA barcoding; this species was then identified using the *Ace*2 gene, which was shown to share between 99.6% and 100% identity with GenBank (MN299021; MK575480) (Table 3).

**Table 3:** Details of the result of the specimens submitted to molecular biology analysis and homology with the reference sequences available on GenBank using BLAST

Mosquito species (morphological ID)	No. sequenced	Percentage of similarity between sequences of same species	Molecular identification (% identity)	Accession no.
-Specimens using for confirming MALDI-TOF MS identification				
*Ae. aegypti*^a^	3	100%	*Ae. aegypti* (100%)	MN299016
*Ae. albopictus*^a^	3	99.7–100%	*Ae. albopictus* (99.7–100%)	MN299017, MK995332
*Cx. quinquefasciatus*^b^	3	99–100%	*Cx. Quinquefasciatus* (99.6–100%)	MN299021, MK575480
*Cx. sitiens*^a^	2	100%	*Cx. sitiens* 99.8%	MF179212
*An. aconitus*^a^	2	99.8%	*An. aconitus (*100%)	HQ877378
*An. annularis*^a^	1	99.5%	*An. annularis* (96.6%)	KF406655
*An. dirus*^a^	2	100%	*An. dirus* (99.7%)	JX219732
*An. minimus* s.l^a^	2	100%	*An. harrisoni* (99.8–100%)	HQ877377
*An. minimus* s.l.^a^	2	100%	*An. minimus* (99.4%)	HQ877337
*An. jamessi*^a^	2	89.2–100%	*An. jamessi* (99.7–99.8%)	MT380518, MT871938
*An. pediteaniatus*^a^	3	96–99.5%	*An. pediteaniatus* (99.3–99.8%)	LC333267
*An. sinensis*^a^	2	100%	*An. sinensis* (99.7%)	MG816536
*An. vagus*^a^	2	100%	*An. vagus *99.7%	MK685245
*An. maculatus*^a^	3	100%	*An. maculatus* 99.8%	KT382822
*An. varuna*^a^	2	100%	*An. varuna* (100%)	HQ877380
Specimens used for database update				
*An. aconitus*^a^	2	90.4–99.8%	*An. aconitus* (99.8–100%)	HQ877378
*An. annularis*^a^	1	-	*An. annularis* (96.6%)	KF406655
*An. dirus*^a^	3	89.7–100%	*An. dirus* (99.5–99.7%)	JX219732
*An. minimus* s.l.^a^	4	90–99.84%	*An. harrisoni* (99.8–100%)	HQ877377
			*An. minimus* (99.4%)	HQ877337
*An. barbirostris*^a^	1	99.7%	*An. barbirostris* (99.7%)	AB971312
*An. jamessi*^a^	4	89.2–100%	*An. jamessi* (99.7–99.8%)	MT380518, MT871938
*An. pediteaniatus*^a^	4	96–99.5%	*An. pediteaniatus* (99.3–99.8%)	LC333267
*An. sinensis*^a^	1	98.7–98.9%	*An. sinensis* (99.5–99.7%)	MG816536, MG816562, KX779641
*An. vagus*^a^	3	98.2–98.7%	*An. vagus *(99–99.7%)	MK685245, MH425442, MF179262
*An. maculatus*^a^	2	100%	*An. maculatus* 99.8%	KT382822
*An. varuna*^a^	1	-	*An. varuna* (100%)	HQ877380
*An. splendidus*^c^	2	99.8%	*An. splendidus* (98.4–98.7%)	MK685253
*Culex* spp.^a^	15		*Cx. tritaeniorhynchus* (99.3–100%)	MF179219, MF179221, MF179231, MF179232
			*Cx. fuscocephala* (100%)	HQ398887
			*Cx. pseudovishnui* (97.9–98.2%)	AB738092, LC054483
			*Cx. pallidothorax* (98%)	MF179281
			*Cx. vishnui* (99.2%)	MF179240
Specimens with discrepancies between morphological and MALDI-TO MS identification				
*An. aconitus*^a^	1	-	*An. varuna* (100%)	HQ877380
*An. dirus*^a^	1	-	*An. sinensis* (99.5%)	MG816562
*An. minimus*^a^	4	99–100%	*An. varuna* (99–100%)	HQ877380
*An. jamesi*^a^	1	-	*An. maculatus* (93.8%)	KT382822
*An. pediteniatus*^a^	2	99.5%	*An. sinensis* (99.4–99.5%)	MG816562, KX779641
*An. maculatus*^a^	9	100%	*An. maculatus* (93.6%)	KT382822
*Culex* sp.^a^	1	-	*An. minimus* A (99.5%)	HQ877337
*Culex* sp.^a^	1	-	*An. jamesii* (99.7%)	MT871938
*Culex* sp.^a^	1	-	*An. dirus* (99.7%)	JX219732

^a^*COI*_1 gene

^b^*Ace2* gene

^c^*COI*_2 gene

Similarly, for specimens that showed discrepancies between our morphological identification and MALDI-TOF MS identification, i.e., *An. aconitus* identified as *An. varuna* by MALDI-TOF MS, *An. dirus* identified as *An. sinensis*, *An. minimus* identified as *An. varuna*, *An. jamesi* identified as *An*. *maculatus*, *An. pediteniatus* identified as *An. sinensis*, and *Culex* sp. identified as *An. minimus*, *An. jamesii*, and *An. dirus*. We found consistent identification between MALDI-TOF MS and molecular tools after querying the sequences against the GenBank database. However, for some specimens that were morphologically identified as *An. maculatus* s.l., the *COI*_1 sequences were 93.44% identical to *An. maculatus* (KT382822). We had no sequence for these specimens with the *ITS* gene generally used to differentiate between species of the *An. maculatus* complex (Table 3).

Finally, interrogation of the sequences from the samples selected for confirmation showed a perfect match between MALDI-TOF MS and molecular identification (Tables 2 and 3).

**Fig. 4:**
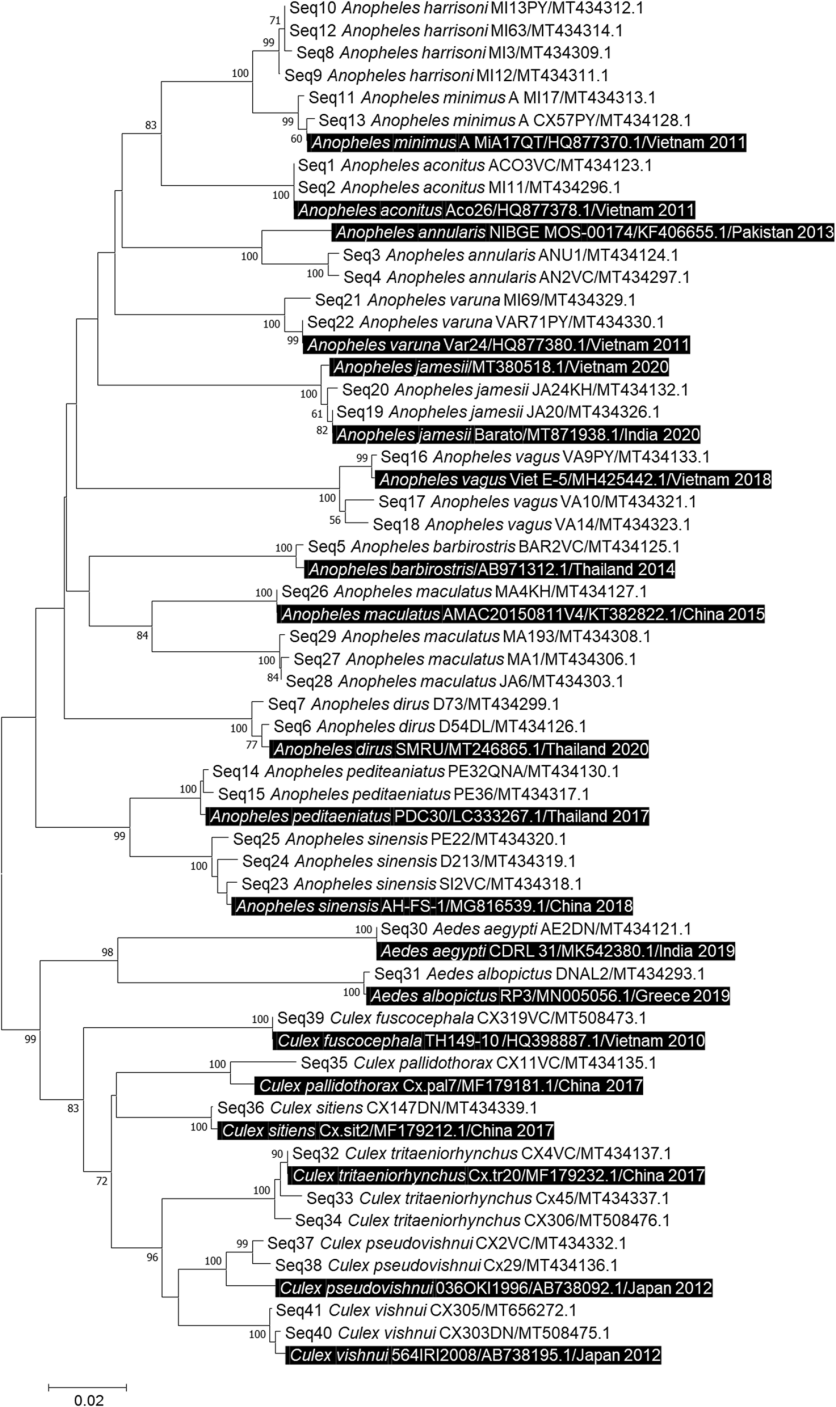
Neighbour-joining tree of genetic relationships obtained from *COI*_1 DNA barcode from 20 mosquito species collected in Vietnam (not including *Anopheles splendidus* by *COI*_2 and *Culex quinquefasciatus* by *Ace*2 gene) compared to other reference sequences (highlighted in dark text) in the GenBank database based on the 720-bp partial *COI*_1 gene. For the sequences downloaded from the GenBank database, the species names are indicated with their GenBank accession numbers and the country of origin, followed by the year they were deposited in GenBank. Node numbers are percentages of bootstrap values

The *COI*_1*, COI*_2 or* Ace2* sequences of some specimens were deposited in GenBank, and the names and accession numbers are listed in Additional file 5: Table S3. The phylogenetic tree constructed using the *COI_*1 sequences and their homologues available on GenBank shows that these 22 mosquito species formed distinct groups (Fig. 4) while correlated with morphological and MALDI-TOF MS identifications.

## Discussion

MALDI-TOF MS is an innovative tool for the rapid and accurate identification of arthropods [21]. Here, we report the first MALDI-TOF MS reference spectra of a selection of protein extractions from Vietnamese mosquito legs. These species are widely distributed, medically important mosquitoes, and were most frequently captured during entomological surveillance and management programmes in the Central Highlands of Vietnam. Among them, *An. minimus* s.l., *An. dirus* s.l. and *An. maculatus* s.l. are considered to be the main vectors of human malaria in Vietnam [40–42] and other parts of Southeast Asia, such as Laos, Cambodia and Thailand [28, 43, 44]. *Aedes albopictus* and *Ae. aegypti*, are vectors of DENV, and recent epidemics of dengue fever in 2017 and 2019 resulted in 183,287 and 200,000 cases, with 32 and 50 deaths, respectively [45–47]. These two *Aedes* species are also important vectors of the CHIKV and Zika virus, both of which are present in the arbovirus burden of disease in Vietnam [48]. Mosquitoes of the genus *Culex* are the main vectors responsible for the transmission of JEV throughout Vietnam [49]. Despite the introduction of a JEV vaccine in 1997 and mandatory vaccination of children under the age of 5 years in Vietnam [49] over the past 10 years, the average annual number of JEV cases is between 1000 and 1200, with 20 to 50 deaths [50].

In terms of entomological surveillance, all mosquito species collected using the four methods in this study did not really have much of an impact on the quality of the MALDI-TOF MS profile, despite the duration of exposure to the mosquitoes according to temperature and humidity varying widely. After 12 h of use of the animal-baited net trap, the CDC-LT and the BG-sentinel trap, compared to the HLC method, the LSV median values remained at the optimal threshold score (i.e. 1.7). The stability of the MALDI-TOF MS results could likely be explained by the mosquitoes being handled carefully before being stored in dry tubes with silica gel.

Two species of *Aedes* and 14 species of *Anopheles* were identified based on morphological characteristics. In contrast, *Culex* specimens were only identified to the genus level based on morphological features due to the integrity of the mosquitoes, which did not allow for discrimination between morphologically identical sibling mosquito species and cryptic species groups. Although morphological identification is the “gold standard” for distinguishing mosquitoes based on external characteristics, it is unable to separate species belonging to the same complex. In the present study, almost all of the *Culex* species that were identified belong to species complexes that are not only difficult to distinguish morphologically [51] but also molecularly [23, 39, 52].

High-quality MS spectra were obtained for 84% of mosquito samples analysed by MALDI-TOF MS. The quality of the spectra obtained from *Aedes* spp. was higher than that obtained for MS spectra from *Anopheles* spp. and *Culex* spp. However, our percentage of good-quality spectra was lower than that found in a study of mosquitoes stored at − 20 °C [53]. This is due to the difference in storage methods, as demonstrated by previous studies which have highlighted that storage at − 20 °C is the best method of preserving arthropods for MALDI-TOF MS analysis for periods not exceeding 6 months [54] while for MALDI-TOF MS analyses that are to be performed after periods exceeding 6 months, the best method of storage is − 80 °C [39]. However, many laboratories do not have a − 80 °C freezer for mosquito storage, and in these cases − 20 °C freezing is the optimal method. In our study, conducted under field conditions, the percentage of good-quality spectra was higher than that of another study on mosquitoes which were also preserved in silica gel [20].

The MS spectra generated from the legs of the different mosquito species and the dendrogram show intra-species reproducibility and inter-species specificity or grouping of specimens of the same species on the same branch (Fig. 2; Additional file 2: Figure S1). This specificity of the MALDI-TOF MS spectra of mosquitoes has already been reported [20, 23, 53].

MALDI-TOF MS analysis allowed us to identify 22 species of mosquitoes, with LSV scores ranging from 1.708 to 2.843, including 18 species that were not in our MALDI-TOF MS database. Two species of *Anopheles* (*An. kochi* and *An. jeyporiensis*) were identified based on morphological features and 11 specimens of *Culex* sp. were not identified by MALDI-TOF MS because their spectra were not in our database. The latter is due to *An. kochi* and *Culex* not being molecularly identified in our study because we did not obtain DNA sequences from them, despite having good-quality spectra. An arthropod spectrum is added to our database only when the specimen is unambiguously identified by morphology and molecular biology analysis [17]. We had only one specimen of *An. jeyporiensis* for which the MS spectrum was of poor quality [lower intensity: < 3000 AU; which was non-reproducible and which contained background noise). In addition to being able to identify closely related mosquito species, we found that MALDI-TOF MS can distinguish between subgroups of species of the same complex, which are impossible to distinguish morphologically, as was the case for the *An. minimus* complex (*An. minimus* and *An. harrisoni* formerly referred to as *An. minimus* A and C) [55, 56] and for the *An. maculatus* s.l. complex (Figs. 2, 3). Interestingly, the first two top hits for species classification were *Cx. quinquefasciatus* and *Cx. pipiens*, both ranging from 99 to 100% of sequence correspondence. These *Cx. quinquefasciatus* specimens were then classified by *Ace*_2 (Table 3). Preliminary studies had already reported that the MALDI-TOF MS technique is capable of identifying arthropods at the species, species subgroup or complex level [20, 57].

The morphological identification of all the specimens introduced in our database was confirmed by molecular biology using at least one gene. We found consistency between our morphological and molecular identifications, as our sequences had identities ranging from 96.6 to 100% with their GenBank homologues. For specimens that showed inconsistencies between morphological identification and MALDI-TOF MS identification, molecular biology analysis agreed with the MALDI-TOF MS identification. Morphological identification errors thus occurred, showing the limitations of this method. These errors were partly identified by MALDI-TOF MS, which we consider to be reliable for the identification of arthropods. Sequencing, which is the last technique we used to either confirm morphological and MALDI-TOF MS identification or to separate the two, did not allow us to identify 11 *Culex* specimens for which we had no morphological and MALDI-TOF MS identification of the species. This shows that all techniques have limitations and that molecular identification requires a reliable genomic sequence and the presence in GenBank of the homologous sequences of the specimen to be identified [26]. Despite the difficulties and limitations of morphological identification, it remains the gold standard when it comes to identifying an unknown specimen, especially when the molecular method cannot identify it due to the absence of its sequence in GenBank or due to the lack of a sequence.

## Conclusion

This study is the first to apply MALDI-TOF MS to the identification of field-caught mosquitoes in Vietnam, where the burden of disease transmitted by these vectors is high. It confirms that misidentification of mosquitoes by morphology can be prevented with MALDI-TOF MS, provided that the reference spectrum of that mosquito species is present in the MALDI-TOF MS database. The study allowed us to enrich our in-house MALDI-TOF MS database by adding reference spectra of new species. MALDI-TOF MS is a promising entomological surveillance tool that can significantly contribute to the control of mosquito-borne diseases in Vietnam.

## Supplementary Information

**Additional file 1: Table S1.** Distribution of the adult mosquito species collected in different regions of Vietnam.

**Additional file 2: Figure S1.** Comparison of MALDI-TOF MS spectra from the legs of 22 mosquito species collected in Vietnam between May 2018 and January 2020. MS spectra revealed intra-species reproducibility and inter-species specificity. Abbreviations: a.u., Arbitrary units; m/z, mass to charge ratio

**Additional file 3: Table S2.** List of the top ten average mass peaks of the different mosquito species. The top ten average mass peaks by mosquito species are shown in bold. The average intensity of the mass peaks is indicated using a colour scale from green to red, indicating moderate and high average peak intensity, respectively. The list of mass peaks used to distinguish different mosquito species is based on the analysis of the ClinProTools genetic algorithm model. Abbreviations: Da, Daltons

**Additional file 4: Figure S2.** Flow diagram of mosquito specimens which were included, analysed, and added to our in-house database using MALDI-TOF MS and molecular tools

**Additional file 5: Table S3.** Accession numbers of sequences of different species of mosquitoes deposited in GenBank

## Data Availability

All relevant data are presented in the paper and its supporting ESM files. Our MALDI-TOF MS database is publicly available on our laboratory website and can be downloaded with the following DOI number: https://doi.org/10.35081/hwtr-5224.

## Abbreviations

BG-Sentinel: Biogents Sentinel mosquito trap
BLAST: Basic Local Alignment Search Tool
CDC-LT: US Centers for Disease Control and Prevention miniature light traps
ID: Identification
LSVs: Log score values
MALDI-TOF MS: Matrix-assisted laser desorption/ionization time-of-flight mass spectrometry
