# Supplementary material for: MALDI-TOF mass spectrometry identification of mosquitoes collected in Vietnam
Source: Parasit Vectors. 2022 Jan 28;15:39. doi: 10.1186/s13071-022-05149-2 (PMC8795957; doi:10.1186/s13071-022-05149-2)
Supplement: Supplementary file 1 — Additional file 1: Table S1. Distribution of the adult mosquito species collected in different regions of Vietnam. [file 13071_2022_5149_MOESM1_ESM.docx]

**Additional file 1: Table S1. Distribution of the collected adult mosquito species in different regions of Vietnam**

| **Collection sites** | **Latitude/longitude coordinates** | **Species** | **Total** |
| --- | --- | --- | --- |
| **Da Nang** | 16.03 N 108.01 E | *Ae. albopictus*, *Ae, aegypti*, *Culex* spp. | 902 |
| **Quang Nam** | 15.39N 107.30 E | *Ae. aegypti*, *Ae. albopictus*, *An. maculatu*s, *An. minimus* s.l, *An. splendidus*, *An. aconitus*, *An. peditaeniatus*, *An. sinensis*, *An. kochi*, *Culex* spp. | 220 |
| **Binh Dinh** | 13.37 N 108.59 E | *Ae. aegypti*, *Ae. albopictus*, *An. maculatus*, *An. minimus* s.l, *An. peditaeniatus*, *An. jamesii*, *An. splendidus*, *An. vagus*, *An. aconitus*, *An. sinensis*, *An. varuna*, *An. kochi*, *An. barbirostris*, *An. annularis*, *Culex* spp. | 1098 |
| **Phu Yen** | 13.22 N 109.02 E | *Ae. aegypti*, *Ae. albopictus*, *An. maculatus*, *An. dirus*, *An. minimus* s.l,  *An. peditaeniatus*, *An. splendidus*, *An. vagus*, *An. aconitus*, *An. barbirostris*,  *An. annularis*, *Culex* spp. | 899 |
| **Gia Lai** | 13.15 N 108.45 E | *An. maculatus*, *An, dirus*, *An. peditaeniatus*, *An. splendidus*, *An. barbirostris*, *An. jeyporiensis*, *Culex* spp. | 473 |
| **Dak Lak** | 12.49 N 108.27 E | *An. maculatus*, *An. dirus*, *An.peditaeniatus*, *An. splendidus*, *An. vagus*,  *An. barbirostris*, *Culex* spp. | 233 |
| **Khanh Hoa** | 12.16 N 108.53 E | *An. maculatus*, *An. dirus*, *An. splendidus*, *An. vagus*, *An. aconitus*,  *An. sinensis*, *Culex* spp. | 209 |
| **Binh Thuan** | 11.09 N 108.03 E | *An. maculatus*, *An. dirus*, *An. minimus* s.l, *An. peditaeniatus*, *An. vagus*,  *An. varuna*, *Culex* spp. | 181 |
| **8 provinces** | | **24 species** | **4215** |

Lat_Lon: Latitude/longitude coordinates; DN: Da Nang; QN: Quang Nam; BD: Binh Dinh; GL: Gia Lai; DL: Dak Lak; KH: Khanh Hoa; BT: Binh Thuan
