# Supplementary figures and images for: MALDI-TOF mass spectrometry identification of mosquitoes collected in Vietnam
Source: Parasit Vectors. 2022 Jan 28;15:39. doi: 10.1186/s13071-022-05149-2 (PMC8795957; doi:10.1186/s13071-022-05149-2)

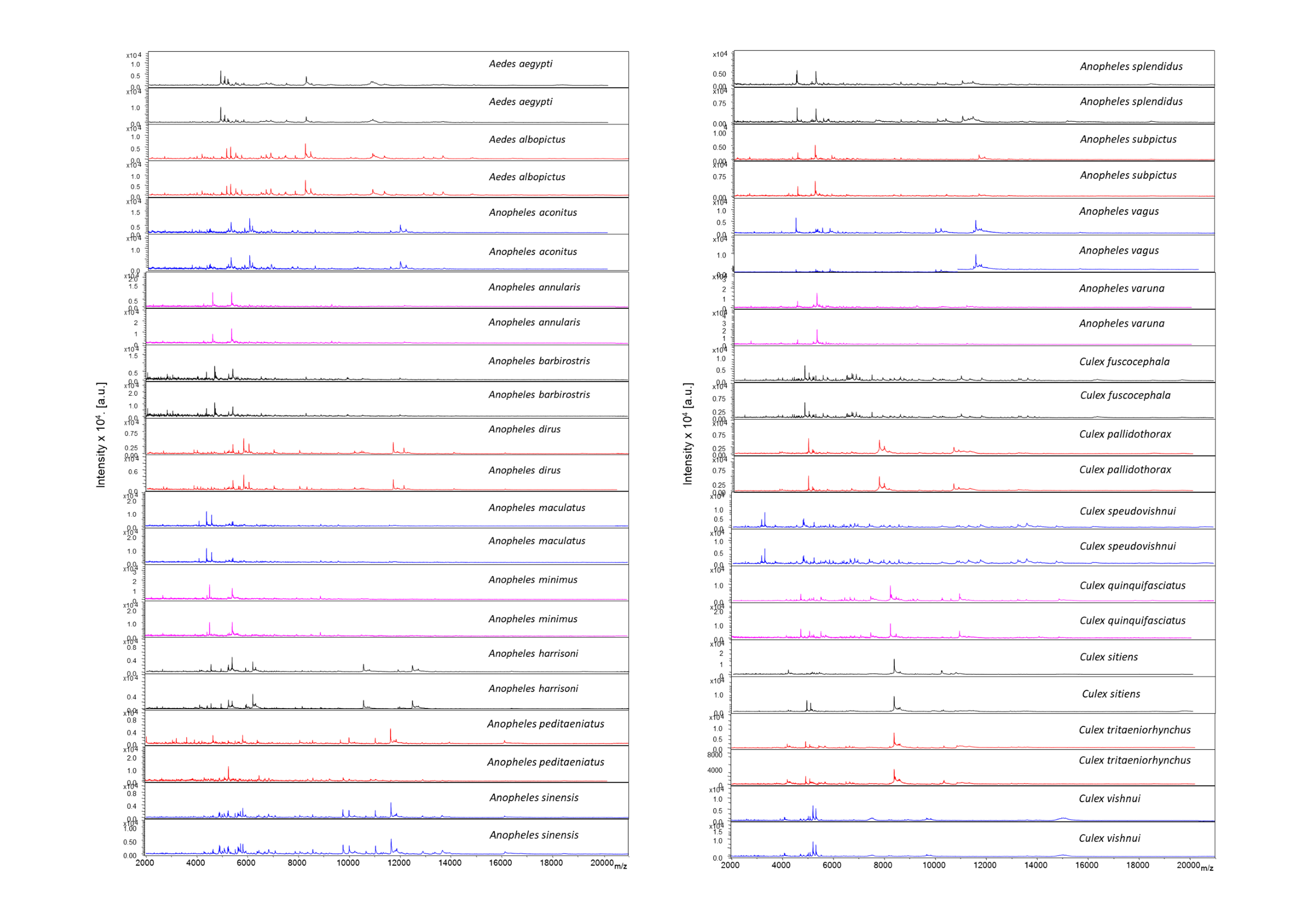

Supplement: Supplementary file 2 — Additional file 2: Figure S1. Comparison of MALDI-TOF MS spectra from the legs of 22 mosquito species collected in Vietnam between May 2018 and January 2020. MS spectra revealed intra-species reproducibility and inter-species specificity. Abbreviations: a.u., Arbitrary units; m/z, mass to charge ratio [file 13071_2022_5149_MOESM2_ESM.tif]

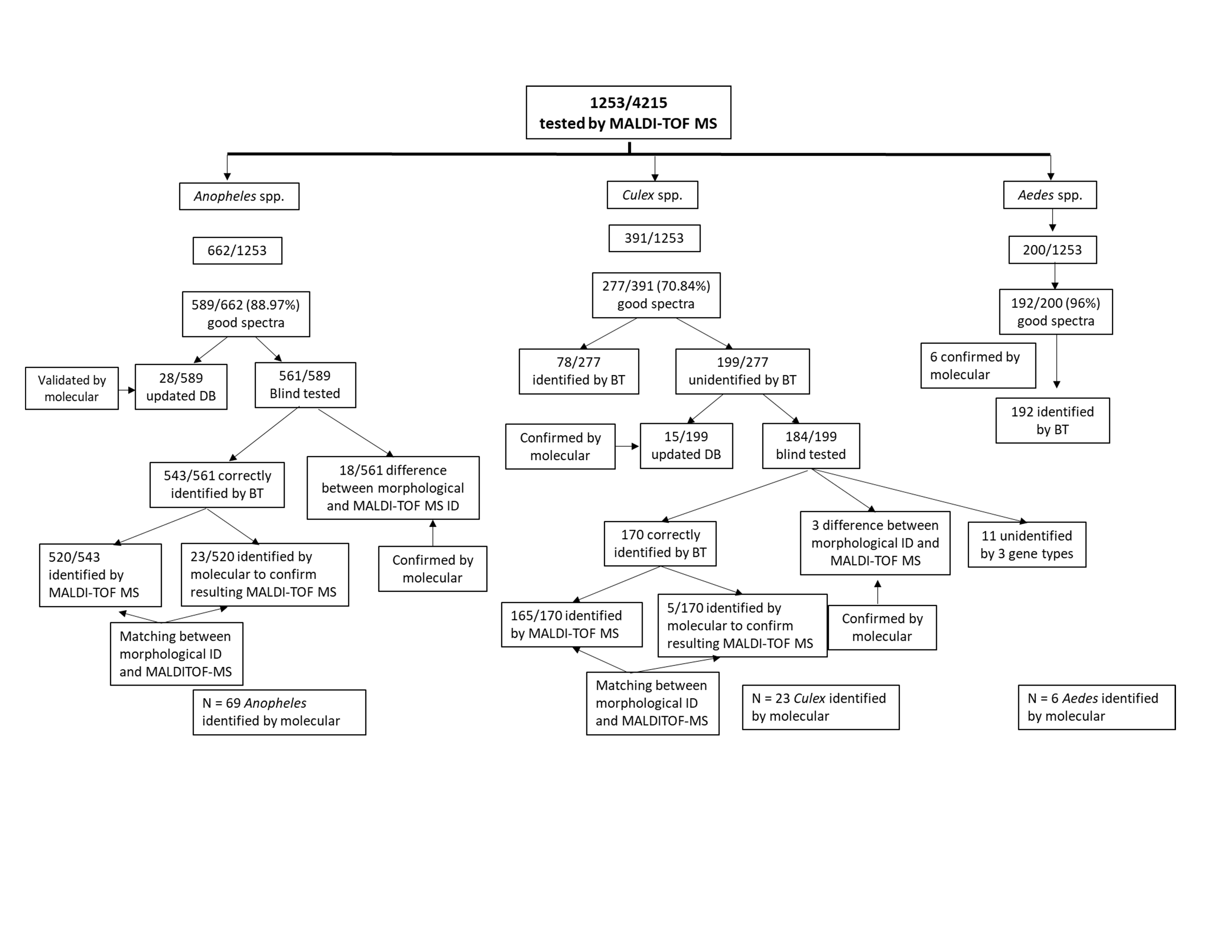

Supplement: Supplementary file 4 — Additional file 4: Figure S2. Flow diagram of mosquito specimens which were included, analysed, and added to our in-house database using MALDI-TOF MS and molecular tools [file 13071_2022_5149_MOESM4_ESM.tif]
