# Supplementary material for: MALDI-TOF mass spectrometry identification of mosquitoes collected in Vietnam
Source: Parasit Vectors. 2022 Jan 28;15:39. doi: 10.1186/s13071-022-05149-2 (PMC8795957; doi:10.1186/s13071-022-05149-2)
Supplement: Supplementary file 5 — Additional file 5: Table S3. Accession numbers of sequences of different species of mosquitoes deposited in GenBank [file 13071_2022_5149_MOESM5_ESM.docx]

**Additional file 5: Table S3.** Accession numbers of sequences of different species of mosquitoes deposited on GenBank

| **Species** | **Gene** | **Accession number(s)** |
| --- | --- | --- |
| *Aedes aegypti* | *COI*_1 | MT434121, MT434122, |
| *Aedes albopictus* | *COI*_1 | MT434292, MT434293, MT43494 |
| *Anopheles aconitus* | *COI*_1 | MT434123, MT434295, MT434296 |
| *Anopheles annularis* | *COI*_1 | MT434124, MT434297 |
| *Anopheles barbirostris* | *COI*_1 | MT434125 |
| *Anopheles dirus* | *COI*_1 | MT434126, MT434298, MT434299, MT434300 |
| *Anophels maculatus* | *COI*_1 | MT434127, MT434305 |
| *Anopheles minimus* A | *COI*_1 | MT434128, MT434313 |
| *Anopheles minimus* C | *COI*_1 | MT434129, MT434309, MT434310, MT434311, MT434312, MT656272 |
| *Anopheles peditaeniatus* | *COI*_1 | MT434130, MT434315, MT434316, MT434317 |
| *Anopheles sinensis* | *COI*_1 | MT434131, MT434319, MT434320 |
| *Anopheles splendidus* | *COI_*2 | MW425609, MW425610 |
| *Anopheles jamesii* | *COI*_1 | MT434132, MT434324, MT434325, MT434326, MT508472 |
| *Anopheles vagus* | *COI*_1 | MT434133, MT434321, MT434322, MT434323 |
| *Anopheles varuna* | *COI*_1 | MT434134, MT434327, MT434328, MT434329, MT434330, MT434331, MT656275 |
| *Culex pallidovishnui* | *COI*_1 | MT434135 |
| *Culex speudovishnui* | *COI*_1 | MT434136, MT434332, MT434335 |
| *Culex tritaeniorhynchus* | *COI*_1 | MT434137, MT434333, MT434334, MT434336, MT434337, MT508476, MT656273 |
| *Culex sitiens* | *COI*_1 | MT434138, MT434338 |
| *Culex fuscocephala* | *COI*_1 | MT508473, MT508477, MT508478 |
| *Culex vishnui* | *COI*_1 | MT508475, MT656270, MT656271, MT656272 |
| *Culex quinquefasciatus* | *Ace*2 | MW449856, MW449857, MW449858, MW449859, MW449860 |
